# Contrast Sensitivity Testing in Healthy and Blurred Vision Conditions Using a Novel Optokinetic Nystagmus Live-Detection Method

**DOI:** 10.1167/tvst.10.12.12

**Published:** 2021-10-06

**Authors:** Peter Essig, Yannick Sauer, Siegfried Wahl

**Affiliations:** 1 Institute for Ophthalmic Research, University of Tübingen, Tübingen, Germany; 2 Carl Zeiss Vision International GmbH, Aalen, Germany

**Keywords:** optokinetic nystagmus, contrast sensitivity, psychophysics, eye movements

## Abstract

**Purpose:**

The aim of the current study was to develop and validate an automated contrast sensitivity (CS) test using a live- detection of optokinetic nystagmus (OKN) and an adaptive psychometric procedure. In addition, the study sought to replicate the known effect of defocus on CS for the OKN-based measurements in emmetropic participants.

**Methods:**

Fifteen participants viewed a horizontally moving grating while their eyes were tracked with an infra-red (IR) eye-tracker. To simulate the clinical conditions of the CS measurements, the participants were stimulated monocularly as the left eye was occluded by an IR filter. The horizontal eye position was continuously analyzed for OKN responses, and the stimulus contrast was changed by an adaptive psychometric method depending on the outcome. Furthermore, the newly proposed OKN live-detection was verified against an offline analysis and an expert-observer judgement. The OKN-based CS was measured for six spatial frequencies at normal vision and three levels of defocus using spherical convex lenses.

**Results:**

The newly proposed OKN live-detection method showed a sufficient detection performance for implementation of adaptive procedures, and the detection rate is similar or better compared to offline detection methods. Spatial frequency and defocus had a significant effect on the OKN-based CS (*P* < 0.0001 for both).

**Conclusions:**

The current study presents a novel method to measure motion CS in an automated way, combining the real-time detection of OKN and an adaptive psychometric procedure. Furthermore, the known effect of defocus on CS was successfully replicated with the newly developed tool.

**Translational Relevance:**

OKN-based CS is a novel approach to assess spatial vision, which is sensitive to subtle effects of defocus, allowing use with nonverbal patients and infants. Furthermore, the newly developed tool may improve the performance of such measurements.

## Introduction

Assessment of contrast sensitivity (CS) from eye movements has already been proposed as a possible method to gain objective information. Moreover, these measurements may help examine noncommunicative participants.^1^ Previous research has shown that in objective testing of CS, several types of eye movements can be implemented, namely microsaccades^2^^–^^4^ or smooth pursuit eye movements.^1^ In addition, the eye movement occurring in response to a moving scenario, optokinetic nystagmus (OKN), has been linked to the appraisal of CS (motion CS).^5^^–^^7^ Canonically, OKN is a saw-tooth displacement of the eye, denoting the two phases of the OKN. The slow phase (OKN-SP) identifies a motion-tracking eye movement occurring in the direction of the visual stimulus drift. This phase is similar in nature to smooth pursuit eye movements, because largely overlapping neural circuitry was found in fMRI measurements.^8^ Furthermore, the velocity of the OKN-SP appears to be lower, but nonetheless comparable to the velocity of the moving pattern, because the OKN gain (the ratio of the OKN-SP velocity to the stimulus velocity) was found to be 0.76 ± 0.15 using EOG.^6^ In contrast, the quick phase of OKN (OKN-QP) occurs in the direction against the stimulus drift in a saccade-like fashion, moving the eye into the original position. It has been previously believed that the OKN-QPs are similar to normal saccades, because the main sequence parameters have not revealed any statistical difference between saccades and OKN-QPs, although the velocity of an OKN-QP was found to be slightly lower.^9^ However, recent research revealed that the OKN-QPs are not triggered by an attentional input, and therefore the OKN-QP should not be considered as a classical saccade.^10^ Despite this, the detection of an OKN-QP is possible using the velocity-based algorithms initially proposed for saccadic (microsaccadic) detection.^11^ Furthermore, two types of OKN can be differentiated based on the initial instructions provided to an observer: stare-OKN, in which case the participant is required to attempt to fixate a limited area on a screen, and look-OKN, where the participant tries to follow the stimulus and, hence, pay attention. Although the execution of stare-OKN failed to activate cortical oculomotor centers significantly, it was suggested that the look-OKN demands more higher-level neural processing.^8^ In agreement with the study by Dakin et al.,^5^ we used the stare-OKN paradigm in the current study. With regard to OKN and visual performance appraisal, OKN appeared to be a reliable eye movement in assessment of visual functions in participants suffering from several visual impairments.^6^ Although previous research focused on detecting visual acuity from OKN returned results that correlated with subjective measurements in both youth and adults,^12^^,^^13^ the estimation of visual acuity in children using OKN reflexes did not show sufficient performance.^14^ Regarding the testing of contrast sensitivity, Sangi et al.^15^ showed an efficient assessment of CS in children, as did Leguire et al.^7^ in emmetropic adults.

In addition, Dakin et al.^5^ showed highly correlated CS curves obtained by OKN responses and actual responses of the participants to moving noise patterns, implicating OKN responses as a potentially useful tool in CS measurements. Nonetheless, OKN has been identified either by subjective judgements or using offline detection algorithms after the measurement was conducted. The approach of searching for the OKN contrast threshold offline suffers from poor time efficiency, because it prevents the implementation of adaptive procedures that adjust stimulus parameters while performing the experiment. Some of the current algorithmic OKN-detection methods could be adapted to OKN live detection, as suggested by Mooney et al.^1^; however, they deemed the existing methods of OKN detection to be insufficient. Therefore we aimed to develop a real-time OKN detection method with sufficient detection performance that allows the application of adaptive procedures for time-efficient and automated OKN-based measurements of CS. In this method, the contrast level was sought over a selected range of spatial frequencies at which the OKN response just occurs and therefore extends the applicability of OKN into clinical domain. Here, for performing the search of the contrast thresholds, we used the QUEST+ adaptive psychometric procedure.^16^ The used psychometric function used was the Weibull function, which is used in both clinical^17^ and OKN-based^5^ CS measurements. Furthermore, replication of the known effect of defocus on CS has not been tested in OKN-based CS measurements. Hence, the new tool was used to measure OKN CS under normal vision, as well as in three conditions of defocused vision using convex spherical lenses. Because contrast thresholds have been shown to shift toward higher contrast levels with increasing defocus mainly for visual stimuli of higher spatial frequencies in clinical measurements of CS using various stimuli,^18^^–^^20^ the current study was targeted to obtain a similar effect in the OKN-based CS measurements. The results of the OKN-based CS being influenced by defocus serves as an additional verification of OKN as a tool for CS measurements. After the clinical measure of CS, testing was performed under monocular stimulation. The left eye was covered by an IR filter that still allowed binocular eye tracking. This method did not show any significant change in accuracy of the video-based eye tracking.^21^

## Methods

### Participants

Fifteen participants in a mean age of 24.7 ± 3 (four male and 11 female), participated in the current study. All participants were emmetropic. The current study considered emmetropia as a refractive error of smaller than ±0*.*5 *D* in spherical equivalent obtained by the wavefront-based autorefraction (ZEISS i.Profiler plus; Carl Zeiss Vision, Aalen, Germany) in their tested (right) eye. Furthermore, all participants had a negative history of ocular, systemic, or neurological disease, amblyopia, or trauma. The study protocol followed the Declaration of Helsinki. In addition, the study was approved by the ethics committee of the Faculty of Medicine of the University Tuebingen, and signed informed consent was obtained from all participants before the experiment. All participants were recruited from the University of Tuebingen.

### Visual Stimulus and Eye Tracking

For triggering OKN, we used a vertically oriented square-wave grating drifting over the horizontal plane with a constant velocity of *v* = 2.3° s^−1^, as used in the previous studies^6^^,^^11^^,^^22^^,^^23^ in OKN-based visual performance measurements. Because no clear effect of OKN gain has been found between the two horizontal directions,^24^ the grating was moved either nasally or temporally in an equal number of trials, in a random order. The stimuli were created in MATLAB (MATLAB2018b; MathWorks, Natick, MA, USA) using Psychtoolbox-3^25^^,^^26^ and were covering the entire Viewpixx screen (VIEWPixx; VPixx Technologies Inc., Saint Bruno, Quebec, Canada). Because the screen provided a resolution of 1920 × 1200 pixels with a pixel pitch of 0*.*252 mm, the covered visual field from the viewing distance 75 cm was ∼36° and ∼23° in the horizontal and vertical planes, respectively. Furthermore, the screen provided a gray-scale bit depth of 12 bits, whereas the luminance nonlinearity was corrected via gamma correction. Here the mean luminance of the screen was 130 cd/m^2^. The refresh rate was 120 Hz. The selected spatial frequencies (*SF*s), calculated for the observing distance of 75 cm, were *SF* = 0*.*7*,* 1*.*5*,* 2*.*6*,* 3*.*7*,* 5*.*2*,* 6.5 cycles per degree (cpd). The order of measured *SF*s was randomized for every defocus condition. The contrast of the stimulus for each trial was selected from 39 available contrast levels ranging from ∼0.03% to ∼66%. The motion of the stimulus of a given contrast level was aborted at *t* = 4 s after stimulus onset or immediately after a robust OKN response was detected by the live analysis, making the testing as time-efficient as possible. The number of trials per *SF* was set to a constant value of 64, giving a comparable amount of data for all tested conditions. After every presentation of the stimulus, a gray cross of 1*.*25° in size appeared for *t* = 1*.*3 s. Participants were asked to blink during the presentation of this irrelevant stimulus, whereas the contrast level for the next trial was defined by QUEST+, running in the background. Eye tracking was performed using the EyeLink 1000 Plus eye tracker (SR Research, Ontario, Canada) with a fixed sampling rate of 1000 Hz. To measure CS under monocular condition, the left eye was covered by an IR filter (ePlastics, San Diego, CA, USA) with a transmission of T > 90% for λ > 800 nm, allowing tracking of both eyes while presenting the stimulus only to the right eye. Before every measurement, a nine-point calibration procedure of the eye tracker was performed.

### Live OKN Detection

In the current study, we propose a new method for OKN live detection during stimulus presentation. This approach in combination with an adaptive psychometric procedure allows the searching for a contrast threshold in an automated and time-efficient way. Here, the sampling and consequent analysis of gaze data was coupled to the refresh rate of the of the screen (120 Hz). For the OKN-QP detection, we used a modified version of Engbert's velocity-based algorithm^27^ with the model's free parameter λ = 7. Noise-level calculation of both eyes was performed just over the horizontal plane from the gaze data over the first 34 frames (283 ms) of every presentation of the grating. This time period was used only for the noise assessment. To reduce the computational demand, only horizontal gaze data was evaluated and Engbert's algorithm was applied to data samples from the last detected saccade. The binocular overlap criterion for saccadic detection was used, as originally proposed by Engbert et al.,^27^ as the gaze position was captured for both eyes.

**Figure 1.**
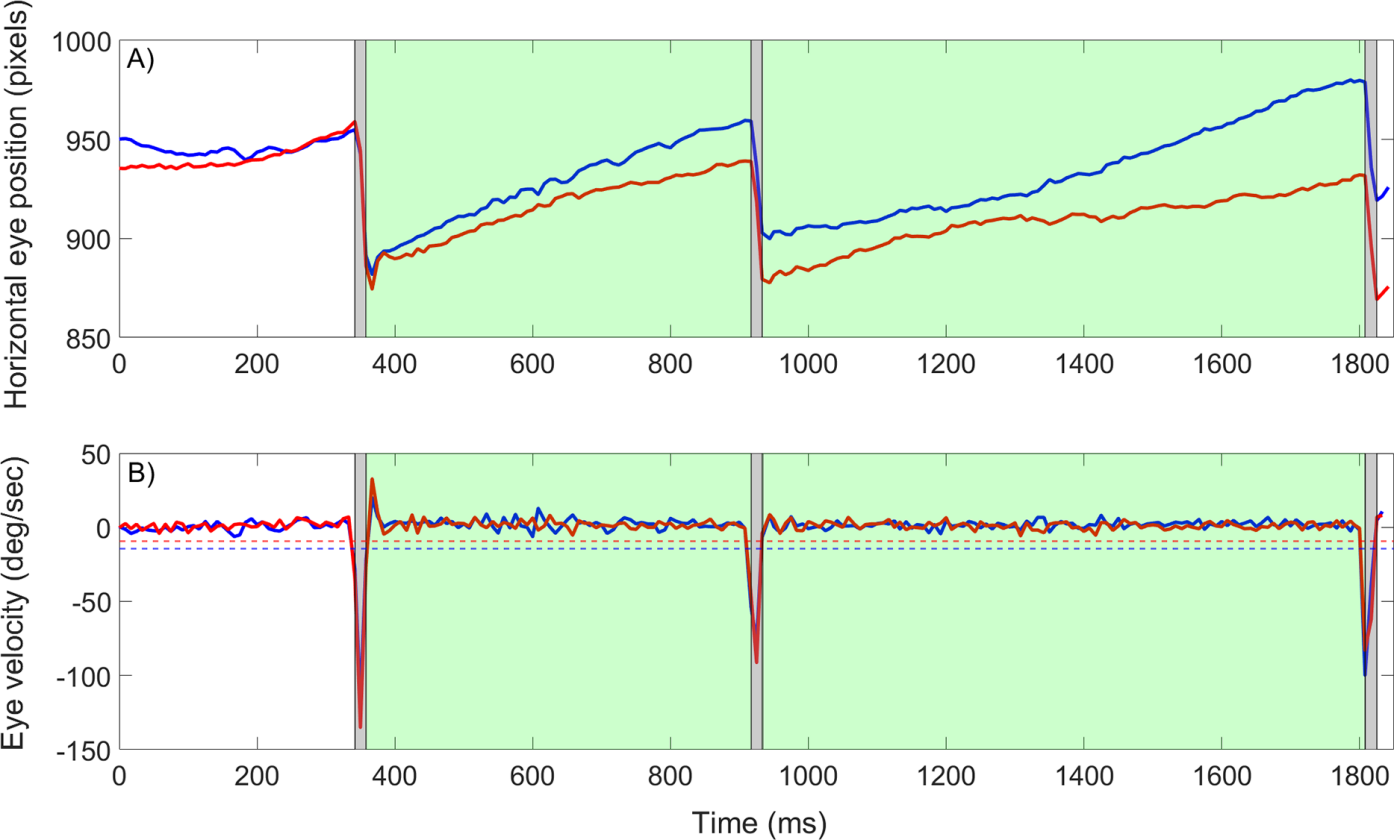
Horizontal eye position for both eyes (A) and derived velocity (B) during one trial with OKN. In both figures the *gray areas* denote the limits of detected saccades (OKN-QP), followed by a *green area* for a successfully detected OKN-SP. In panel B, the noise threshold for saccade detection is depicted in *blue* for the left eye and *red* for the right eye. Note that with the maximum time for grating presentation set to 4 s, this trial was aborted after ≈ 1.8 s, because a robust OKN was detected with the live detection method.

For the detection of OKN-SP, the time frame between the end of any first saccade and the start of a subsequent saccade was identified, as an OKN-SP appears between two OKN-QPs, as shown in the Figure 1. Whenever the start of a second saccade was detected, the horizontal gaze data samples were analyzed considering a potential OKN-SP. In the next step, the direction and the mean velocity of the OKN-SP were calculated, with a prior smoothing over five data samples using a moving average filter in MATLAB. Moreover, the analyzed data was shortened by adding (subtracting) ∼17 ms (two refreshments of the screen) to the start (end) of a saccade, to avoid saccadic overshoot influencing the OKN-SP velocity calculation. Last, the sign of the velocity was compared with the direction of the moving stimulus, as only OKN events that occurred in the direction of the moving stimulus were taken into consideration. Furthermore, we required a robust OKN response to a given stimulus. Therefore, similarly to Turuwhenua et al.,^28^ at least two OKN events must have been detected to consider the stimulus as seen, as depicted in Figure 1.

The performance of the newly developed OKN detection algorithm, coupled to the screen refresh rate (120 Hz), was compared to offline analysis of the same measurements with the full sampling frequency of 1000 Hz. Both the online and offline OKN detection procedures were compared with the judgement of an experienced observer. In the expert rating, the first author (P.E.) affirmed whether a correctly oriented robust OKN response (two OKN events) could be seen in the gaze data. For the offline OKN detection, we used the original version of Engbert's velocity-based algorithm^27^ for OKN-QP detection, with the model's free parameter λ set to 7 and the minimum time difference between two saccades set to 50 ms to prevent overshoots from being detected as separate eye movements. In contrast to the live detection method, the offline analysis included blink detection. Here the blinks were detected as missing pupil events. These events were removed with a buffer of *t* = 50 ms to protect the data from blink-related artefacts. The OKN detection in both live and offline method relied on the OKN-SP velocity thresholding.

### Experimental Procedure

The current study considered the implementation of an adaptive psychometric algorithm as an efficient method to estimate CS from OKN in real time. Hence, we used the QUEST+ algorithm^16^ to change the contrast level depending on whether an OKN response was detected, to return the threshold contrast for OKN. As a psychometric function, the Weibull function was used with the parameters threshold and slope. Upper asymptote (lapse rate) and lower asymptote (guess rate) were set to zero. The parameter space for threshold had 39 contrast levels. Because Dakin et al.^5^ showed that the slope can vary across subjects and parameters of the visual stimulus, the slope also had a predefined parameter space of 0*.*5 to 5*.*5 in steps of 0*.*5. Furthermore, because the duration for convergence was expected to vary among subjects, the number of the grating presentations was fixed, resulting in comparable amounts of data for every participant.

Moreover, although Dakin et al.^5^ showed that OKN can be used to objectively assess CS, there is limited evidence that decreased CS can be reliably measured as corresponding OKN decrements if defocus is present. Hence, we systematically evaluated the effects of defocus, which decrease VA and CS, on the OKN-based CS. To study this effect, we introduced three convex lenses of different optical power in fine steps, +1.5 D, +2.0 D, and +2.5 D. These lenses were inserted in a trial frame in a random order with a fixed vertex distance of 12 mm. We considered the defocus-induced magnification to be negligible, given its low values below 5%.^29^ The viewing distance of the screen was *d* = 75 cm, leading to an accommodational demand of 1.33 D. The lenses were selected to have the optical power above this demand.

### Data Analysis

To find the OKN contrast threshold, OKN occurrence rate dependent on stimulus contrast level was fitted with a psychometric function using Psignifit^30^ in MATLAB. Here, the cumulative Weibull distribution function was used as the fitting function with the following parametrization

(1) $$\;\;\;\begin{array}{c} \Psi \left(x;m,w,\gamma ,\lambda \right)=\gamma +\left(1-\lambda -\gamma \right)\left(1-e^{\log\left(0.5\right)e^{c\frac{\log(x)-m}{w}}}\right) \end{array}$$

with *c* = log(−log(0*.*05)) − log(−log(0.95)). Guess rate γ and lapse rate λ, representing the lower and upper asymptote, were both set to zero. The threshold *m* and the width *w*, defining the zone between the 0*.*05 and 0*.*95 points, are in log space of the stimulus parameter *x*. The relationship between the width *w* and the slope *s* of the Weibull function is $s=\frac{c}{w}$. The contrast threshold *CT* was calculated from *m* as *C* = *e^m^*, at which the OKN occurs with a probability of 50 %. This threshold was taken for every measured spatial frequency of the grating and was later converted to a CS value as $CS\;=\;\frac{1}{CT}$.

The contrast sensitivity function (CSF) was created as a fit of the CS values depending on spatial frequency *SF* with a log-parabola, considering the ascending and descending part of the CSF as already suggested by Lesmes et al.,^31^ or more recently by Dakin et al.^5^ using the following fitting function:

(2) $$\;\;\;\begin{array}{c} CS\left(SF\right)=\log_{10}\left(\gamma_{\max}\right)-\log_{10}\left(2\right){\left(\frac{\log_{10}\left(SF\right)-\log_{10}\left(SF_{\max}\right)}{\beta }\right)}^{2}, \end{array}$$

with fitting parameters *γ_max_*, *SF_max_* and *β*. These denote the peak sensitivity, the peak spatial frequency and the function's bandwidth, respectively.^31^ The statistical analysis tested the effect of spatial frequency and defocus level on contrast sensitivity using a repeated measures two-way analysis of variance. The data has been tested for its normality on a default level of significance 5% in MATLAB. We evaluated the goodness of fit of the proposed log-parabola CSF with the coefficient of determination for the various conditions of defocus.

## Results

### Evaluation of the OKN Detection Performance

**Figure 2.**
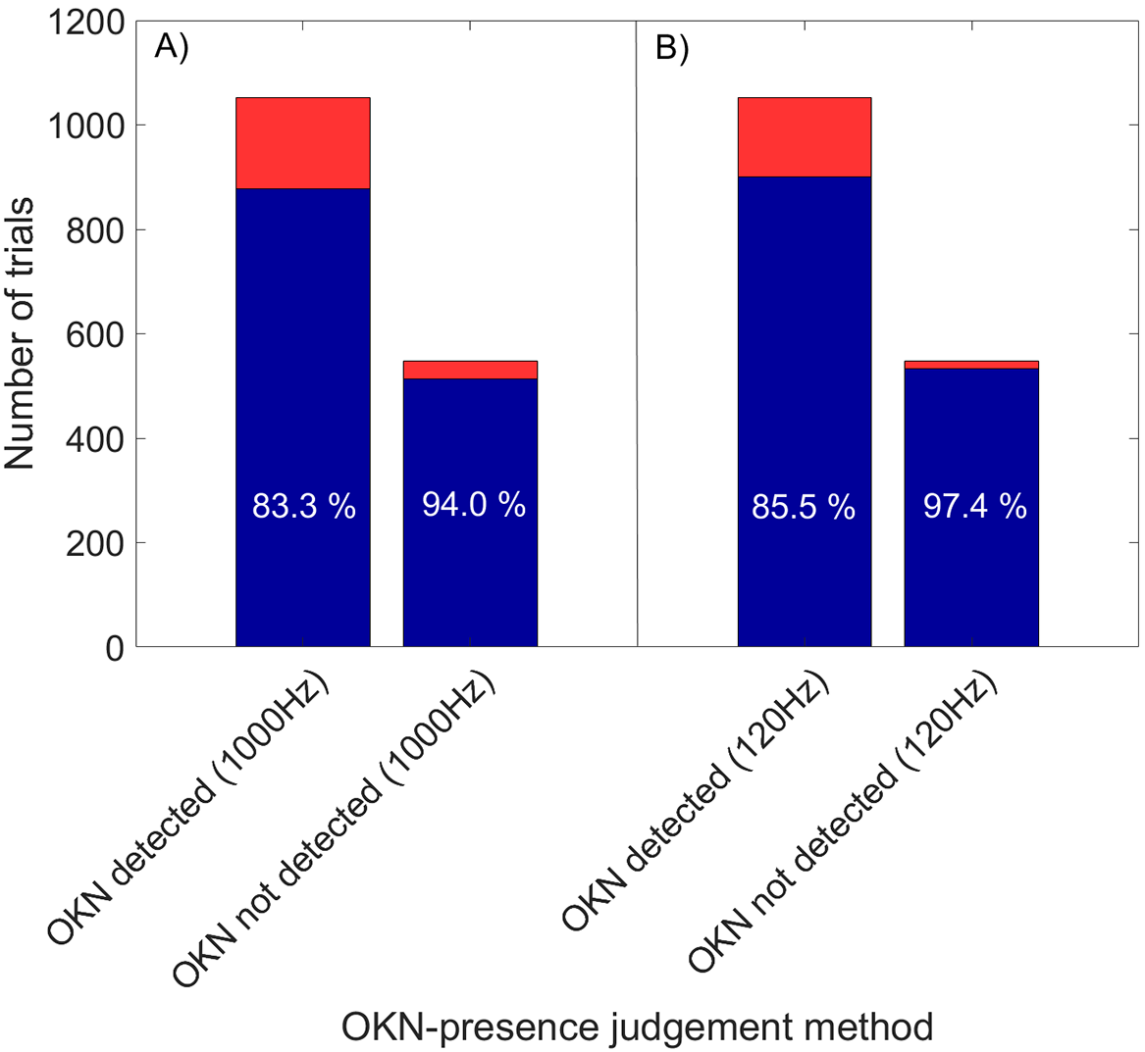
Two approaches of OKN analysis are compared to the subjective judgment of OKN presence across 1600 tested trials. These approaches were the offline post-measurement analysis using the original sampling rate of the eye tracker—1000 Hz, and the newly proposed live OKN analysis limited by the screen refresh rate of 120 Hz. The amount of trials (not) containing the robust OKN response are given by the first (second) of the two groups (A and B). The first bar in both groups shows the true-positive (*blue*) and the false-negative (*red*) proportion similarly to the second bar showing the true-negative (*blue*) and false-positive (*red*) OKN detection, respectively.

The newly proposed OKN live detection algorithm was designed to abort the trial after two valid OKN events, as shown in Figure 1. Here, the performance of the live detection was evaluated against a subjective judgement of an experienced observer and the offline post-analysis of the data with original sampling rate of the eye tracker, as shown in Figure 2. For this analysis, 1600 trials were randomly selected from the whole study data set, covering trials of all subjects, defocus conditions and spatial frequencies. Both approaches showed decent performance in OKN detection in trials of actual presence of robust OKN response by correctly identifying 83*.*3% and 85*.*5%, using the offline and live detection method, respectively. Furthermore, these algorithms showed nearly excellent classification of trials of no actual OKN response, because a correct judgement was found in 94*.*0% and 97*.*4% for the respective approaches. These results show a successful application of the live tracking in OKN detection procedure.

### Contrast Sensitivity Revealed by OKN With and Without Induced Defocus

**Figure 3.**
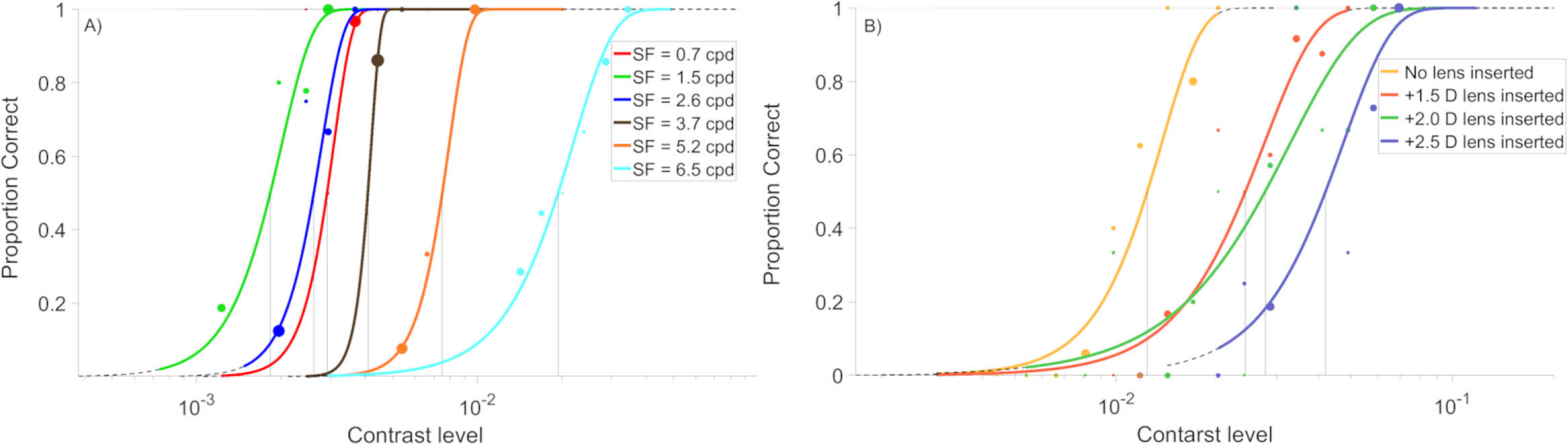
(A) The six Weibull fits correspond to the measurements of the six spatial frequencies within one defocus condition (here the fits shown were obtained for the highest defocus level in one selected participant). (B) The four Weibull fits represent the measurements of the four conditions of defocus within one spatial frequency (here the fits shown were obtained for the *SF* = 3*.*7 cpd in one selected participant). The *black lines* always target the contrast level, at which the OKN response is estimated to occur with the probability of 50%. Dot size scales with the testing frequency of the particular contrast level, as selected by the adaptive psychometric procedure.

**Figure 4.**
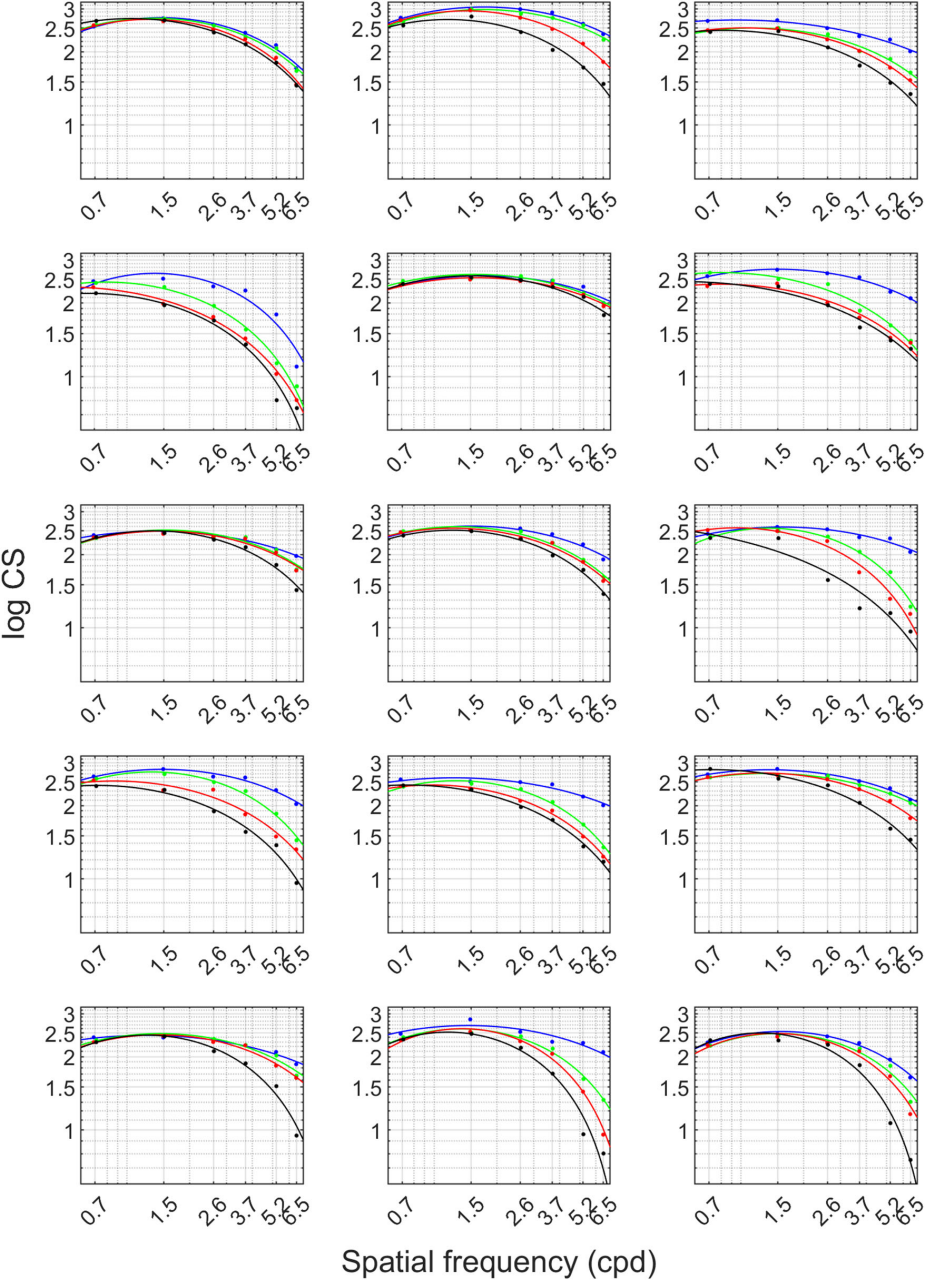
The figure contains fifteen panels, each representing data for an individual participant. The four curves in every panel show the estimated CSF for every measurement condition. The *blue curve* always represents natural viewing condition of the tested emmetropes, the following *green line* represents the first step in defocus (+1.5 D lens inserted in the trial frame), continuing with the *red (black) curve* obtained for the viewing conditions through the +2.0 D (+2.5 D) lens, respectively.

The CS values were obtained from the Weibull psychometric fits of the OKN proportion detected across a range of tested contrast levels. These were acquired for each of the six tested spatial frequencies, as shown in Figure 3A for one typical subject. As depicted in panel (A) of Figure 3, the smallest contrast threshold (the best CS), is given for the green fit, obtained for a grating of 1*.*5 cpd, later resulting in the peak of the CSF, followed by the blue and red fit for gratings of 2.6 cpd and 0.7 cpd, respectively, giving the CSF the log-parabolic shape. Last, the brown, orange, and cyan fits show the results for the gratings of 3*.*7 cpd, 5*.*2 cpd, and 6*.*5 cpd in spatial frequency, showing the already observed trend of decreasing CS with increasing *SF*. In panel (B), the four Weibull fits are provided from measurements of healthy vision and the three defocus steps for the highest used spatial frequency, 6*.*5 cpd in one selected subject. Finally, the CSFs were fitted for each defocus condition and clustered for every subject, as shown in Figure 4. The time needed to perform a single measurement of CS for one given spatial frequency, considering the number of trials, the set cross-presentation time, and the velocity of the stimulus, ranged between four and five minutes, depending on the defocus condition.

The proposed log-parabola curve used for fitting the CS values over a range of spatial frequencies showed a goodness of fit of *R*^2^ > 0.84 in every condition. Further statistical testing, using repeated measures two-way analysis of variance, showed a significant effect of both spatial frequency (F(5,340) = 188.98; *P* < 0.0001) and defocus level (F(3,342) = 52.98; *P* < 0.0001). Furthermore, the interaction of the two independent variables effect showed a strong significance (F(15,330) = 19.16; *P* < 0.0001). Thus the known effect of defocus decreasing the ability to detect a contrast pattern, as well as a disclosure of CSF created for a wide range of spatial frequencies, was successfully replicated with the newly developed tool.

## Discussion

Obtaining information about patient's visual performance in an objective way using eye movements has become an area of interest for many researchers.^1^^–^^5^^,^^14^^,^^21^^,^^32^ The current study followed the finding that OKN responses may serve as a reliable tool for assessment of CS^5^ and extended the applicability by using an automated search method for the contrast threshold, based on a live evaluation of eye-tracking data. Moreover, the replication of the clinically-known effect of defocus on CS, already shown by Marmor et al.,^18^ Green et al.,^19^ or similarly by Jansonius et al.^20^ in edge-CS measurements, were not sufficiently tested in OKN-based CS measurements. Hence, we aimed to replicate the consequence of defocus with the newly developed tool in emmetropic subjects.

We used a live OKN detection and an adaptive psychometric method to measure CS by searching for the contrast threshold at which the OKN just occurs for a given spatial frequency. As an adaptive psychometric method, we used the QUEST+ algorithm, because this procedure was advised to be used in CS testing.^16^ We implemented the algorithm in its one-dimensional form for the contrast level management; however, the implementation could be extended by using a multidimensional psychometric function on spatial or temporal frequency to increase the speed of the assessment of the spatiotemporal CSF. The OKN detection performance of the newly proposed method showed sufficient accuracy compared to the subjective judgment and very similar performance to the offline algorithmic-based analysis, therefore allowing the implementation of an adaptive psychometric procedure. However, not all trials were detected correctly. Some trials were found to contain a robust OKN response in the correct direction but have not been detected by the proposed algorithm, or vice versa. A possible reason for this is the limitation of not having a blink identification in the live OKN detection procedure. A second limitation is that the noise level has been estimated across a quite short time period, because the time performance of the measurements was privileged over an extended detection time. Third, some trails, especially those in which a low-contrast-grating was presented, may have contained OKN-SPs of a velocity below the defined threshold, whereas the experienced observer recognized it still as a valid OKN-SP. Such algorithmic misjudgment may have happened because the detection algorithms used a fixed threshold value for the OKN-SP velocity. As already shown in the zebrafish experiment by Rinner et al.,^33^ the gain of OKN (ratio of the OKN-SP velocity to the physical velocity of the stimulus) varies across contrast levels and is lower for low-contrast stimuli. Hence, we suggest instead thresholding the OKN-SP according to the contrast level, which could be implemented in the future. Nonetheless, the method of OKN detection we used in the current study was found to be superior to previous works, because the false-positive rate of existing detection algorithms was judged as too high for the implementation of adaptive methods.^1^ The CS value was obtained from the Weibull fit, already used in the previous research,^5^ because the inverted value of the contrast level at which the OKN response was expected to occur with a probability of 50 %. Furthermore, the assessment of the log-parabolic fit, which was already proposed in the previous work by Lesmes et al.,^31^ showed robust goodness of fit in the trend of CS values over selected spatial frequencies. Here, the CSF's peak is shifted toward smaller spatial frequencies, compared to the CS curves obtained in clinical practice in which nonmoving stimuli are used. However, this effect has already been observed in the previous study.^34^ Moreover, the current study results show an agreement of the CSF-peak placement with the previous study by Burr et al.,^34^ considering the stimulus velocity used in each experiment. As a next point, the current study aimed to measure CS under healthy and blurred vision conditions in several steps, and thus replicate the known effect of decreasing CS with increasing defocus,^18^^–^^20^ mainly having an impact on gratings of higher spatial frequencies. Such testing is possibly done in two ways, once by decreasing vision to a certain value of visual performance, as done by Marmor et al.,^18^ or by using an optical power of a lens resulting in comparable refractive error (defocus) across subjects.^32^ Since the current study tested emmetropes, lenses of constant values have been used over all participants to artificially worsen their vision. Here, the defocus levels have been chosen to induce the desired blur, although with negligible simultaneous magnification-induced effects on the presented spatial frequencies.^29^ Hence, lenses in the range from +1.5 D to +2.5 D in +0.5 D steps were used. Because the current study targeted clinical testing, similar to previous work,^32^ we did not use cycloplegic agents to suppress accommodation. Furthermore, we avoided cycloplegia because these substances result in pupil dilation, leading to increasing high-order aberrations and decreasing the CS. As the results show decreasing CS to increasing defocus, mainly for higher spatial frequencies, the current study successfully replicated the effect of defocus on CS in OKN-based measurements. Possible reasons for the variation in the impact of defocus are the residual refractive error of the emmetropic participants, their status of high-order aberrations, or differences in lags of accommodations, because these factors are highly individual.^35^^,^^36^ At the last point, since the current study used a square-wave grating to enable displaying also gratings of high spatial frequencies, the effect of higher harmonics might be comprised in the data. As shown by Campbell et al.^37^ and Graham et al.,^38^ based on Fourier theory the detection threshold for a mixture of sine-waves is the determined by the component that reaches its contrast threshold first. In our case, for a square-wave of a base frequency *SF* = 1*.*5 cpd or higher, the first-order harmonic (three times the base frequency) is *SF* = 4*.*5 cpd or higher. Given that the detection thresholds for this spatial frequencies are beyond the peak of the CSF, only the higher harmonics of low-spatial-frequency square-wave gratings might be considered as relevant. Nonetheless, because no subject showed a peak CS that was three times higher than the one at about *SF* = 0*.*7 cpd in the previous study using a sine-wave grating,^34^ it appears that the participants detected the square wave gratings by its fundamental *SF* and not by one of the higher harmonics. In summary we consider the effect of higher harmonics on our OKN-based CS in our selected range of spatial frequencies to be negligible.

## Conclusion

In conclusion, the current study successfully tested CS with the newly developed tool in a clinical environment over various conditions of healthy and blurred vision. We found that the proposed OKN live detection method is accurate enough for the usage with an adaptive psychometric procedure, estimating the participant's CS in an automated and time-efficient way. Furthermore, this study showed a successful replication of the blur effect on CS measured with the newly developed tool. Hence, the current study indicates the possibility to use OKN to assess visual performance for non-communicative patients, not only considering CS but also visual acuity or visual field loss, as recently suggested.

## References

[1] Mooney SW, Hill NJ, Tuzun MS, Alam NM, Carmel JB, Prusky GT. Curveball: A tool for rapid measurement of contrast sensitivity based on smooth eye movements. *J Vision*. 2018; 18: 7–7. 10.1167/18.12.7 PMC6238984 30452585

[2] Scholes C, McGraw PV, Nyström M, Roach NW. Fixational eye movements predict visual sensitivity. *Proc Royal Soc B: Biol Sci*. 2015; 282: 20151568. 10.1098/rspb.2015.1568 PMC4633872 26468244

[3] Bonneh YS, Adini Y, Polat U. Contrast sensitivity revealed by microsaccades. *J Vis*. 2015; 15: 11–11. 10.1167/15.9.11 26223023

[4] Denniss J, Scholes C, McGraw PV, Nam S-H, Roach NW. Estimation of contrast sensitivity from fixational eye movements. *Invest Ophthalmol Vis Sci*. 2018; 59: 5408–5416. 30452594 10.1167/iovs.18-24674

[5] Dakin SC, Turnbull PR. Similar contrast sensitivity functions measured using psychophysics and optokinetic nystagmus. *Sci Rep*. 2016; 6: 34514. 27698486 10.1038/srep34514 PMC5048294

[6] Wester ST, Rizzo JF, Balkwill MD, Wall C. Optokinetic nystagmus as a measure of visual function in severely visually impaired patients. *Invest Ophthalmol Vis Sci*. 2007; 48: 4542–4548. 17898276 10.1167/iovs.06-1206

[7] Leguire L, Zaff B, Freeman S, Rogers G, Bremer D, Wali N. Contrast sensitivity of optokinetic nystagmus. *Vis Res*. 1991; 31: 89–97. 2006557 10.1016/0042-6989(91)90076-h

[8] Konen CS, Kleiser R, Seitz RJ, Bremmer F. An fmri study of optokinetic nystagmus and smooth-pursuit eye movements in humans. *Exp Brain Res*. 2005; 165: 203–216. 15864563 10.1007/s00221-005-2289-7

[9] Garbutt S, Harwood MR, Harris CM. Comparison of the main sequence of reflexive saccades and the quick phases of optokinetic nystagmus. *Br J Ophthalmol*. 2001; 85: 1477–1483. 11734524 10.1136/bjo.85.12.1477 PMC1723810

[10] Hanning NM, Deubel H. Unlike saccades, quick phases of optokinetic nystagmus (okn) are not preceded by shifts of attention. *J Vis*. 2019; 19: 53c–53c.

[11] Tatiyosyan SA, Rifai K, Wahl S. Standalone cooperation-free okn-based low vision contrast sensitivity estimation in vr-a pilot study. *Restor Neurol Neurosci*. 2020;1–11. 32200360 10.3233/RNN-190937

[12] Schwob N, Palmowski-Wolfe A. Objective measurement of visual acuity by optokinetic nystagmus suppression in children and adult patients. *J Am Assoc Pediatr Ophthalmol Strabismus*. 2019; 23: 272.e1. 10.1016/j.jaapos.2019.05.016 31526857

[13] Millodot M, Miller D, Jernigan ME. Evaluation of an objective acuity device. *Arch Ophthalmol*. 1973; 90: 449–452. 4759430 10.1001/archopht.1973.01000050449008

[14] Cetinkaya A, Oto S, Akman A, Akova Y. Relationship between optokinetic nystagmus response and recognition visual acuity. *Eye*. 2008; 22: 77–81. 16902492 10.1038/sj.eye.6702529

[15] Sangi M, Thompson B, Turuwhenua J. An optokinetic nystagmus detection method for use with young children. *IEEE J Transl Eng Health Med*. 2015; 3: 1–10. 10.1109/JTEHM.2015.2410286 PMC4848063 27170889

[16] Watson AB. Quest+: A general multidimensional bayesian adaptive psychometric method. *J Vis*. 2017; 17: 10–10. 10.1167/17.3.10 28355623

[17] Wallis SA, Baker DH, Meese TS, Georgeson MA. The slope of the psychometric function and non-stationarity of thresholds in spatiotemporal contrast vision. *Vis Res*. 2013; 76: 1–10. 23041562 10.1016/j.visres.2012.09.019

[18] Marmor MF, Gawande A. Effect of visual blur on contrast sensitivity: clinical implications. *Ophthalmology**.* 1988; 95: 139–143. 3344122 10.1016/s0161-6420(88)33218-5

[19] Green D, Campbell F. Effect of focus on the visual response to a sinusoidally modulated spatial stimulus. *JOSA**.* 1965; 55: 1154–1157.

[20] Jansonius N, Kooijman A. The effect of defocus on edge contrast sensitivity. *Ophthalmic Physiol Opt*. 1997; 17: 128–132. 9196675

[21] Essig P, Leube A, Rifai K, Wahl S. Microsaccadic rate signatures correlate under monocular and binocular stimulation conditions. *J Eye Mov Res*. 2020; 13(5). 10.16910/jemr.13.5.3 PMC8008506 33828709

[22] Schober H, Hilz R. Contrast sensitivity of the human eye for square-wave gratings. *JOSA**.* 1965; 55: 1086–1091.

[23] Nachmias J. Effect of exposure duration on visual contrast sensitivity with square-wave gratings. *JOSA**.* 1967; 57: 421–427.

[24] Van den Berg A, Collewijn H. Directional asymmetries of human optokinetic nystagmus. *Exp Brain Res*. 1988; 70: 597–604. 3384058 10.1007/BF00247608

[25] Brainard DH. The psychophysics toolbox. *Spatial Vision*. 1997; 10: 433–436. 9176952

[26] Kleiner M, Brainard D, Pelli D. What's new in psychtoolbox-3? *Perception**.* 2007; 14.

[27] Engbert R, Kliegl R. Microsaccades uncover the orientation of covert attention. *Vis Res*. 2003; 43: 1035–1045. 12676246 10.1016/s0042-6989(03)00084-1

[28] Turuwhenua J, Yu T.-Y, Mazharullah Z, Thompson B. A method for detecting optokinetic nystagmus based on the optic flow of the limbus. *Vis Res*. 2014; 103: 75–82. 25151522 10.1016/j.visres.2014.07.016

[29] Ohlendorf A, Schaeffel F. Contrast adaptation induced by defocus–a possible error signal for emmetropization? *Vis Res*. 2009; 49: 249–256. 19000917 10.1016/j.visres.2008.10.016

[30] Schütt H, Harmeling S, Macke J, Wichmann F. Psignifit 4: pain-free bayesian inference for psychometric functions. *J Vision*. 2015; 15: 474–474.

[31] Lesmes LA, Lu Z.-L, Baek J, Albright TD. Bayesian adaptive estimation of the contrast sensitivity function: the quick csf method. *J Vision*. 2010; 10: 17–17. 10.1167/10.3.17 PMC4439013 20377294

[32] Doustkouhi SM, Turnbull PR, Dakin SC. The effect of refractive error on optokinetic nystagmus. *Sci Reports*. 2020; 10: 1–14. 10.1038/s41598-020-76865-x PMC7676235 33208790

[33] Rinner O, Rick JM, Neuhauss SC. Contrast sensitivity, spatial and temporal tuning of the larval zebrafish optokinetic response. *Invest Ophthalmol Vis Sci**.* 2005; 46: 137–142. 15623766 10.1167/iovs.04-0682

[34] Burr DC, Ross J. Contrast sensitivity at high velocities. *Vis Res*. 1982; 22: 479–484. 7112947 10.1016/0042-6989(82)90196-1

[35] Seidemann A, Schaeffel F. An evaluation of the lag of accommodation using photorefraction. *Vis Res*. 2003; 43: 419–430. 12535999 10.1016/s0042-6989(02)00571-0

[36] Atchison DA, Markwell EL. Aberrations of emmetropic subjects at different ages. *Vis Res*. 2008; 48: 2224–2231. 18639572 10.1016/j.visres.2008.06.023

[37] Campbell FW, Robson JG. Application of Fourier analysis to the visibility of gratings. *J Physiol*. 1968; 197(3): 551–566. 5666169 10.1113/jphysiol.1968.sp008574 PMC1351748

[38] Graham N, Nachmias J. Detection of grating patterns containing two spatial frequencies: A comparison of single-channel and multiple-channels models. *Vis Res*. 1971; 11(3): 251–IN4. 5579840 10.1016/0042-6989(71)90189-1

